# Effect of Beetroot Nitrate Supplementation on Nitric Oxide Pathways and Oxy-Inflammatory Biomarkers in Amateur Triathletes: A Randomized Cross-Over Pilot Study

**DOI:** 10.3390/nu18081215

**Published:** 2026-04-12

**Authors:** Simona Mrakic-Sposta, Alessandra Vezzoli, Mattia Parenza, Marcello Magno, Gennaro D’Angelo, Fabrizio Nannipieri, Santina Battaglia, Linda Solfanelli, Edoardo Tacconi, Cinzia Dellanoce, Michela Montorsi, Lorenza Pratali

**Affiliations:** 1 Institute of Clinical Physiology, National Research Council (IFC-CNR), Piazza dell’Ospedale Maggiore, 3, 20162 Milan, Italy; cinzia.dellanoce@ifc.cnr.it (C.D.); michela.montorsi@uniroma5.it (M.M.); 2 Institute of Clinical Physiology, National Research Council (IFC-CNR), Via Giuseppe Moruzzi 1, 56124 Pisa, Italy; mattia.parenza@yahoo.it (M.P.); magno.marcello@gmail.com (M.M.); gennaro.dangelo@unipi.it (G.D.); lorenza.pratali@cnr.it (L.P.); 3 Medical Research, Abiogen Pharma, Via Antonio Meucci, 36, 56121 Pisa, Italy; faa.nannipieri@gmail.com (F.N.); santina.battaglia@abiogen.it (S.B.); linda.solfanelli@abiogen.it (L.S.); 4 Private Practice, Via Matteo Degli Organi 1, 59100 Prato, Italy; edoardotacconi@me.com; 5 Department of Human Sciences and Promotion of the Quality of Life, San Raffaele Roma Open University, Via di Val Cannuta, 247, 00166 Roma, Italy

**Keywords:** oxy-inflammation, reactive oxygen and nitrogen species, non-professional endurance athletes, beetroot, safety

## Abstract

**Background/Objectives:** Nitric oxide (NO) is a key mediator of vascular, metabolic, and redox pathways, influencing exercise performance. Beetroot, a natural source of inorganic nitrate, increases NO bioavailability and may modulate oxidative stress and inflammation, though data in endurance athletes remain limited. The aim of this study was to assess the effects of a novel beetroot-based nitrate supplement (B-bNs) on NO metabolism, oxidative stress, and inflammation in non-professional triathletes. **Methods:** This was a randomized 2 × 2 cross-over pilot study with two 7-day periods (B-bNs vs. No treatment), separated by a 15-day washout (4 visits: Day 1, 7, 22 and 28). Samples were collected at baseline (T0), 2 h post-first dose (T1), and after 7 days (T2) for the supplementation period (B-bNs) and at T0 and T2 for the “no treatment” period. The following biomarkers from plasma and urine were evaluated: NO pathway (NO metabolites (NOx), nitrite (NO_2_), inducible nitric oxide synthase (iNOS), peroxynitrite, 3-nitrotyrosine (3-NT)), oxidative stress (reactive oxygen species (ROS) production, 8-isoprostane, superoxide dismutase (SOD) activity), and cytokines (IL-6, IL-10). A total of 10 male triathletes (mean age 48.1 ± 9.8 years and BMI 23.9 ± 2.2 kg/m^2^) participated in this study. **Results:** No adverse events were reported. After 7 days of supplementation (T2 vs. T0), significant increases in NOx in plasma and urine (about +155%), iNOS (+56%), peroxynitrite (+60%), 3-NT (+8.6%), ROS (+413%) and IL-6 (+73%) were recorded. These values resulted significantly higher compared to “no treatment” (all *p* = 0.002), with no significant differences for 3-NT, SOD, 8-isoprostane, IL-6, and IL-10. **Conclusions:** Beetroot-based nitrate supplementation may enhance the NO-related pathway in non-professional endurance athletes with nitric-peroxydation activation, occurring without evidence of lipid oxidative damage. Larger placebo-controlled trials with standardized diet/training and performance outcomes are needed to determine the functional significance of these preliminary findings. This study was registered in the ISRCTN registry (ISRCTN10885376).

## 1. Introduction

Nitric oxide (NO) is a key signaling molecule in vascular, metabolic, and muscular physiology [1]. Through the activation of guanylate cyclase, NO induces smooth muscle relaxation and vasodilation, enhancing blood flow to active tissues and reducing oxygen cost during exercise [1]. In skeletal muscle, NO supports mitochondrial biogenesis, contractile efficiency, and metabolic flexibility, thereby sustaining endurance-type performance, particularly in prolonged submaximal efforts [2,3,4]. Importantly, under hypoxic conditions, nitrite (NO_2_^−^) can be reduced back to NO, preferentially in oxygen-deprived tissues, thereby matching perfusion with local metabolic demand [5,6].

Beyond vascular regulation, NO metabolism is closely linked with redox and inflammatory responses. Physiological levels of NO and nitrite can buffer reactive oxygen species (ROS) and reactive nitrogen species (RNS), supporting redox homeostasis [7]. However, concurrent overproduction of NO and superoxide promotes peroxynitrite (ONOO^−^) formation, leading to nitration of proteins and accumulation of 3-nitrotyrosine (3-NT), a marker of nitrosative stress [8]. During exercise, the increased oxygen demand further enhances ROS generation through mechanisms such as ischemia–reperfusion [9], hemoglobin and myoglobin oxidation [10], elevated core temperature, and metabolic byproducts, including catecholamines and lactate [9,10,11]. While moderate ROS levels serve as adaptive signals, excessive production drives lipid peroxidation (8-isoprostane), inflammation, and muscle fatigue [12,13,14]. This redox imbalance is paralleled by immune activation, where cytokines such as interleukin-6 (IL-6, pro-inflammatory) and interleukin-10 (IL-10, anti-inflammatory) reflect the interplay between oxidative stress and inflammatory responses during exercise [15].

Beetroot juice (BJ) is a natural source of inorganic nitrate (NO_3_^−^) generally found in other vegetables or used as preservatives for processed meat products, which undergoes stepwise reduction around 25% to NO_2_^−^ by oral bacteria and when reaches the stomach, some of this NO_2_^−^ is reduced to NO, and subsequently absorbed along with the nonreduced nitrite in the gut passing into the bloodstream where blood NO and NO_2_-concentrations rise [16,17]. Nitrate peaks in plasma about one hour post-ingestion and returns to baseline within 24 h [18]. Increased NO bioavailability has downstream effects, including blood flow dilation through guanylate cyclase mediation, improved muscle blood flow and reducing VO_2_ max at given work rate, glucose uptake, and mitochondrial efficiency [19,20,21].

Due to these properties, BJ has been proposed as both a dietary intervention for cardiovascular and metabolic disorders, such as chronic obstructive pulmonary disease, hypertension, and insulin resistance, and as an ergogenic aid in sports [21,22,23,24,25]. In athletes, several trials and reviews report enhanced endurance capacity, ventilatory efficiency, and exercise tolerance, with benefits most evident in endurance disciplines [26]. Chronic supplementation may also support recovery, attenuating delayed-onset muscle soreness (DOMS) and sustaining repeated performance [27,28]. The heterogeneity of the studies, differences in study design and protocol (nitrate dose, duration, timing of intake in relation to exercise), type of subjects (athletes, sedentary subjects, pathological populations) and outcomes measured make a univocal synthesis difficult [21,22,23,24,25,26,27,28]. Only a minority of investigations have simultaneously assessed biomarkers associated with the NO pathway, oxidative stress and inflammation. Furthermore, individual or variability in response (genetics, baseline oxidative status, training level) can influence who responds positively to supplementation.

Moreover, recent evidence suggests that the ergogenic effects of nitrate supplementation may be attenuated or absent in well-trained athletes, potentially due to already optimized endogenous NO production and physiological adaptations associated with high-intensity training [29,30]. In addition, there is ongoing debate regarding the potential for nitrosative stress or redox imbalance under conditions of high nitrate intake or elevated baseline oxidative status [17,31,32,33]. These considerations highlight the importance of accounting for individual responsiveness when interpreting the physiological and performance-related effects of nitrate supplementation.

Based on this evidence, the present pilot study was designed to evaluate the acute and subacute effects of a beetroot-based nitrate supplement (B-bNs) in amateur male triathletes, a population with high aerobic demands. By simultaneously assessing biomarkers of the NO pathway (NO metabolites; nitrate + nitrite = NOx, inducible nitric oxide synthase; iNOS, peroxynitrite and 3-NT), oxidative stress (ROS, 8-isoprostane, SOD activity), and inflammation (IL-6, IL-10), this study aimed to provide an integrated view of redox and immune adaptations to dietary nitrate supplementation.

In addition, findings from a pilot study investigating the modulation of gut microbiota following two weeks of beetroot juice (BJ) consumption suggest that red beetroot may influence gut microbial composition and related metabolites, with potential health implications [34]. Moreover, according to the available literature, BJ supplementation appears to be safe and not associated with specific adverse effects, except for occasional gastrointestinal discomfort, mainly reported with the consumption of raw beetroot [35]. Therefore, the present study will assess the gastrointestinal tolerability of the product under study according to the Gastrointestinal Symptoms Rating Scale (GSRS) [36].

## 2. Materials and Methods

### 2.1. Study Design and Participants

**Figure 1:**
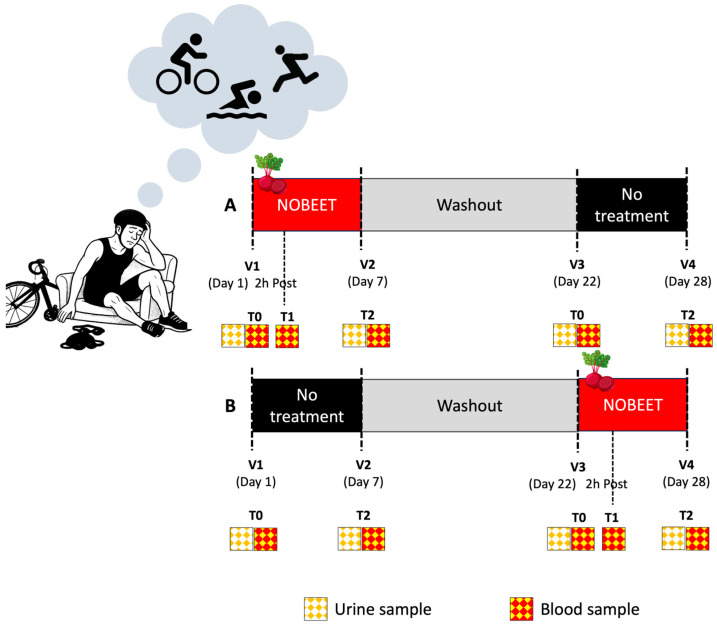
Schematic representation of this randomized 2 × 2 cross-over pilot study. Participants were allocated to one of two sequences: (**A**) NOBEET (B-bNs) for 7 days, followed by a 15-day washout and then a 7-day no-treatment control; or (**B**) 7-day control (No treatment), followed by washout and then 7-day B-bNs. Each subject underwent blood and urine sample collection during four study visits, as illustrated above.

This study was conducted as an open-label, randomized, 2 × 2 cross-over pilot study at the CNR Institute of Clinical Physiology (Pisa, Italy), designed to evaluate the effects of the administration of B-bNs (NOBEET^®^, Gensan, Pisa, Italy) on the NO pathway, oxidative stress, and inflammatory biomarkers in amateur triathletes (Figure 1). Eligible participants were healthy men aged between 30 and 59 years, who regularly practiced non-professional triathlon with at least 300 min of weekly training. All volunteers were required to present a valid medical certification for competitive sports, as well as normal blood pressure and resting heart rate at screening. Participants were admitted to the study only if they had not engaged in competitive races during the four weeks preceding enrollment and had refrained from the use of medications or dietary supplements for at least 14 days prior to study initiation. The experimental phase was conducted during the general preparation period, outside the traditional competitive triathlon season (May to September). This timing allowed participants to be evaluated under stable and controlled physiological load conditions, minimizing the influence of race-related nutritional or training strategies.

Exclusion criteria included smoking, excessive alcohol or caffeine consumption, and the presence of chronic or acute diseases that could interfere with the outcomes of the study. Subjects with known hypersensitivity or allergy to any of the components of the investigational product were excluded. Further, individuals with cardiovascular, metabolic, renal, or hepatic disorders, or those reporting recent infections or inflammatory conditions, were not eligible. To avoid potential confounding from altered physiological responses, subjects with a history of anemia, abnormal hematological parameters, or impaired renal function were excluded. Finally, participants were required to maintain their usual dietary habits and physical activity throughout the study, while any major changes in diet, training load, or recovery strategies during the study period would lead to withdrawal from the analysis.

After screening and informed consent, participants were randomized in a 1:1 ratio into two treatment sequences (Figure 1). Randomization was performed manually, assigning participants to two groups of equal size (*n* = 5 per sequence). Sequence A began supplementation on 30 October 2024, while Sequence B initiated the active phase on 22 November 2024, following the 15-day washout. The 15-day washout period was implemented between intervention phases, which is consistent with the known pharmacokinetics of dietary nitrate and its return to baseline levels within 24 h [17,37]. In sequence A, participants received NOBEET (B-bNs) for 7 days, followed by a 15-day washout and then a 7-day no-treatment control; in sequence B, participants received a 7-day control (No treatment), followed by a 15-day washout and then 7-day B-bNs.

Samples were collected at baseline (T0), 2 h post-first dose (T1), and after 7 days (T2) for the supplementation period (B-bNs) and at T0 and T2 for the “no treatment” period. To minimize circadian variability, all visits were scheduled at the same time of day (±1 h).

All subjects received an explanation of the study purpose, risks, and benefits, read and signed a specific informed consent form. The study design was approved by the National Ethics Committee (A00-ISS-24.04.20204–0018156) on 24 April 2024 and conducted in accordance with the Declaration of Helsinki. This pilot study was retrospectively registered in the ISRCTN registry (ISRCTN10885376; https://www.isrctn.com/ISRCTN10885376) on 23 March 2026.

### 2.2. Treatment

The investigational Nobeet^®^ (Gensan, S.r.l., Pisa, Italy) is a nitric oxide-enhancing supplement supporting both the nitrate-nitrite-NO and L-arginine-NOS-NO pathways. Each 30 g serving provides 0.77 g nitrates from Truebeet™ (*Beta vulgaris*) (Bio-gen Extracts Pvt. Ltd., Bangalore, India), improving blood flow, oxygen delivery, mitochondrial efficiency, and exercise performance [19,38,39,40,41]. L-citrulline (9.9 g) increases arginine availability, enhancing vasodilation, ATP production, and muscular endurance [42]. L-arginine AAKG (3.3 g) further supports NO synthesis, power output, and fatigue delay [42]. Cluster Dextrin^®^ (Gensan, S.r.l., Pisa, Italy) (2.0 g) ensures sustained glucose availability and energy [43]. *N-*acetylcysteine (200 mg) preserves NO bioavailability via antioxidant and nitrate-recycling effects [44].

The daily dose was adjusted according to body weight: subjects weighing 80 kg or less received 10 g of powder dissolved in 100 mL of water, those between 80 and 90 kg received 20 g in 200 mL of water, and those above 90 kg received 30 g in 300 mL of water. The dose of Nobeet^®^ administered to each participant is presented in Table 1. Participants were instructed to take the supplement at the same time each morning to reduce circadian variability, for seven consecutive days during the assigned treatment period. The powder was dissolved in water using a provided shaker to ensure homogeneity and minimize foaming; larger volumes of liquid were permitted if needed for better solubility. Each participant received an individualized product supply for the entire intervention week with a specific measuring cup to obtain the appropriate dosage. To ensure adherence, compliance was monitored through a daily diary completed by participants and by quantifying the amount of returned product at each visit.

### 2.3. Study Visits and Clinical Assessments

Each subject attended four scheduled visits and at each visit, a full medical evaluation was performed, including blood pressure, heart rate, and oxygen saturation (averaged from three consecutive measurements). A baseline echocardiogram was obtained at Visit 1 to exclude subclinical cardiac abnormalities (Vivid I, General Electric Healthcare Clinical System). Body weight and composition were measured using a bioelectrical impedance analyzer (Tanita TBF-300, Tokyo, Japan). Participants also completed the following questionnaires: (i) the short form of the International Physical Activity Questionnaire (IPAQ) to assess weekly activity levels; (ii) a dietary habits questionnaire focusing on nitrate-rich foods; and (iii) the Gastrointestinal Symptom Rating Scale (GSRS) (15 items) to evaluate product tolerability during supplementation weeks [36].

To objectively monitor activity patterns in all subjects, we provided an actigraphy device (Fitbit Inspire 3, Fitbit, San Francisco, CA, USA) for each participant to allow continuous tracking throughout the study.

### 2.4. Sample Collection

For participants who ingested the supplement (B-bNs) on the day of their visit (Sequence A at Day 1 and Sequence B at Day 22), biological samples (i.e., blood and urine) were collected at baseline (T0) and during the acute phase two hours after the first administration (T1). To assess chronic biochemical responses to B-bNs, biological samples were collected after seven days of treatment (T2, Day 7 for Sequence A; Day 28 for Sequence B). Blood and urine samples were collected before (T0, Day 22 for Sequence A; Day 1 for Sequence B) and after seven days (T2, Day 28 for Sequence A; Day 7 for Sequence B) of the no-treatment period too. For each subject, venous blood samples (about 5 mL) were drawn in EDTA and LH tubes (Vacuette tube, Greiner bioone, Kremsmünster, Austria).

The blood samples were centrifuged (Centrifuge 5702 R, Eppendorf, Hamburg, Germany) for 10 min to separate plasma. Plasma samples were collected to determine ROS production, IL-6, IL-10, NO metabolites (NOx), 3-NT, peroxynitrite concentrations, iNOS expression and Superoxide Dismutase (SOD) activity. Urine samples were collected via voluntary voiding in a sterile container provided to the subjects. Urine samples were collected to determine lipid peroxidation (8-iso-PGF2α), NO metabolites (NOx), Nitrite (NO_2_), and creatinine concentration. Samples were aliquoted and stored in a portable Liquid Nitrogen Dewar Supplier during the transport back to the Institute of Clinical Physiology laboratory (IFC-CNR Milan, Italy), then stored at −80 °C until assayed, and thawed only once before analysis.

### 2.5. Biomarker Analysis

#### 2.5.1. ROS Production by Electron Paramagnetic Resonance

Electron Paramagnetic Resonance Spectroscopy (EPR), X-band instrument (E-Scan, 9.3 GHz Bruker Co., Billerica, MA, USA) was used to detect ROS production in the plasma samples, following previously described methods [45,46]. The spin probe CMH (1-hydroxy-3-methoxy-carbonyl-2,2,5,5-tetramethylpyrrolidine), was utilized to enable the acquisition of EPR spectra and the detection of ROS-specific resonances such as superoxide ion, peroxyl radical, peroxynitrite and nitrogen dioxide. The stable radical CP (3-carboxy-2,2,5,5-tetramethyl-1-pyrrolidinyloxy) was then adopted as an external reference to convert the ROS calculated data into absolute quantitative levels (μmol·min^−1^).

#### 2.5.2. Quantification of Inflammatory Markers

IL-6 and IL-10 plasma levels were determined by ultrasensitive ELISA immunoassays (Fine Test, Wuhan, China, Cat No.: EH0201; EH0173), according to the manufacturer’s instructions [47]. The assays are based on a double-antibody sandwich technique. The samples’ concentrations were determined at 450 nm, and plasma levels of inflammatory markers in pg/mL were calculated. The coefficient of variation (CV) was indicated by the manufacturer for IL-6 and IL-10 with inter-assay CV 4.62%, 5.26%, and intra-assay CV 5.35%, 5.05%, respectively.

#### 2.5.3. NO Metabolites

The NO derivatives nitrate and nitrite (NO_2_ + NO_3_ = NOx) were measured in the plasma and urine samples by a colorimetric method based on the Griess reaction, using a commercial kit (Cayman Chemical, Ann Arbor, MI, USA; Item No. 780001), according to the manufacturer’s instructions [47,48]. The samples were read at 545 nm, and the concentration was assessed by a standard curve. The coefficients of variation (CV) indicated by the manufacturer were as follows: inter-assay CV: 3.4%; intra-assay CV: 2.7%.

#### 2.5.4. Inducible Nitric Oxide Synthase (iNOS)

To assess inducible nitric oxide synthase (iNOS) expression in plasma, a human NOS2/iNOS ELISA kit (cat no EH0556; FineTest, Wuhan, China) was used. This assay was based on sandwich enzyme-linked immunosorbent assay technology. NOS2/iNOS protein synthesis was determined using a standard curve. Samples and standards were read at a wavelength of 450 nm, and the analysis was carried out according to the manufacturer’s instructions [47,49,50]. The coefficients of variation (CV) indicated by the manufacturer were as follows: inter-assay CV: 4.89%; intra-assay CV: 5%.

#### 2.5.5. Nitrotyrosine (3-NT)

The concentration of 3-NT in plasma was measured by the competitive-ELISA method, using an assay kit (catalog number EU2560; FineTest, Wuhan, China) according to the manufacturer’s instructions [50]. The 3-NT concentration was measured spectrophotometrically at a wavelength of 450 nm by comparing the sample OD (optical density) to a standard curve. The coefficients of variation (CV) indicated by the manufacturer were as follows: inter-assay CV: 5.68%; intra-assay CV: 6.42%.

#### 2.5.6. Peroxynitrite

Peroxynitrite concentration in plasma was assessed by a commercial kit (MyBioSource, San Diego, CA, USA, Cat. MBS3801965) according to the manufacturer’s instructions [51]. The coefficients of variation (CV) indicated by the manufacturer were as follows: inter-assay CV: 15%; intra-assay CV: 15%.

#### 2.5.7. Superoxide Dismutase (SOD)

SOD activity in plasma was assessed by a commercial kit (Cayman Chemical, Ann Arbor, MI, USA, Cat. 70600) according to the manufacturer’s instructions [52,53]. The coefficients of variation (CV) indicated by the manufacturer were as follows: inter-assay CV: 3.7%; intra-assay CV: 3.2%.

#### 2.5.8. 8-Isoprostane (8-Iso-PGF2α)

Lipid peroxidation was assessed in urine samples using an immunoassay of 8-isoprostane concentration (Cayman Chemical, Ann Arbor, MI, USA, Item No. 516351) [54]. Briefly, 50 μL urine samples were added to a 96-well plate pre-coated with mouse monoclonal antibody, followed by 50 μL of 8-iso PGF2α-tracer antiserum, and incubated. After washing, 200 μL of Ellman’s reagent was added. The coefficient of variation (CV) indicated by the manufacturer was as follows: inter-assay CV 11.5%; intra-assay CV 8.9%.

All the samples and standards were read by a microplate reader spectrophotometer (Infinite M200, Tecan Group Ltd., Männedorf, Switzerland). The determinations were assessed in duplicate, and the inter-assay coefficients of variation were in the range indicated by the manufacturers.

#### 2.5.9. Creatinine

Urinary creatinine concentrations were measured in duplicate using isocratic high-pressure liquid chromatography. The calibration curve was linear (1.25–10 mmol/L). The inter-assay and intra-assay variation coefficients were below 5%. The method has been previously described [54].

Every urinary marker was standardized based on the amount of excreted creatinine, as the collection of the 24 h urine was not possible. In fact, in healthy human subjects in the absence of renal disease, the excretion rate of creatinine remains relatively constant [55].

### 2.6. Sample Size Calculation

An increase in ROS production from 1.68 ± 0.11 to 2.15 ± 0.16 µmol/min^−1^ has been reported following Ironman competition in untreated subjects [56]. However, data on ROS modulation following active supplementation are currently lacking.

The sample size was calculated based on the primary objective of the study: to evaluate the effect of NOBEET supplementation on oxidative stress (ROS) in non-professional male triathletes. Assuming a one-tailed alpha of 0.05, a standard deviation of 0.16, and aiming to detect a 30 percent reduction in ROS, a sample of 10 subjects provides 80 percent power. Accounting for a potential 20 percent dropout rate, 13 participants were planned for enrollment to ensure 10 evaluable subjects.

### 2.7. Statistical Analysis

All statistical analyses were conducted within the cross-over framework, with each participant serving as his own control. Continuous variables were summarized by the number of observations (*n*), mean, standard deviation (SD), and standard error (SE). Shapiro–Wilk testing was utilized to determine the distribution of each data set. Categorical variables were reported as absolute frequencies and percentages. Paired-samples *t*-tests were used for within-subject comparisons when normality assumptions were met, while Wilcoxon signed-rank tests were applied otherwise. Comparisons were performed between baseline and post-supplementation timepoints (T0 vs. T1 and T0 vs. T2), between acute and 7-day values (T1 vs. T2), and between B-bNs and no treatment conditions at day 7. Comparison between levels of physical activity over the study period (by IPAQ or Fitbit) was performed using 1-way ANOVA. A two-sided *p*-value < 0.05 was considered statistically significant. No adjustment for multiple testing was applied, reflecting the exploratory, pilot nature of the study. The confidence interval (CI) for the estimated values was calculated. d-Cohen with 95% CI was used for calculating the size effect. Taking the first measurement T0 as 100%, biological changes were calculated at T1 and T2, allowing an appreciation of the magnitude of change rather than the absolute values. All analyses were carried out using SAS software, version 9.4 (SAS Institute, Cary, NC, USA). Plots were drawn using the GraphPad Prism package for Mac (GraphPad Prism 10.6.1, GraphPad Software Inc., San Diego, CA, USA).

## 3. Results

### 3.1. Participant Demographics and Anthropometrics

All 10 male non-professional triathletes completed the study, and no dropouts were recorded (Group A: *n* = 5, active-first; Group B: *n* = 5, no treatment-first). Table 2 summarizes their baseline characteristics; the BMI recorded by participants was within the normal range. Body composition assessed by bioelectrical impedance analysis confirmed a typical endurance-athlete profile, with low body fat and high fat-free mass percentage, with a total body water of ~65% of body weight.

**Figure 2:**
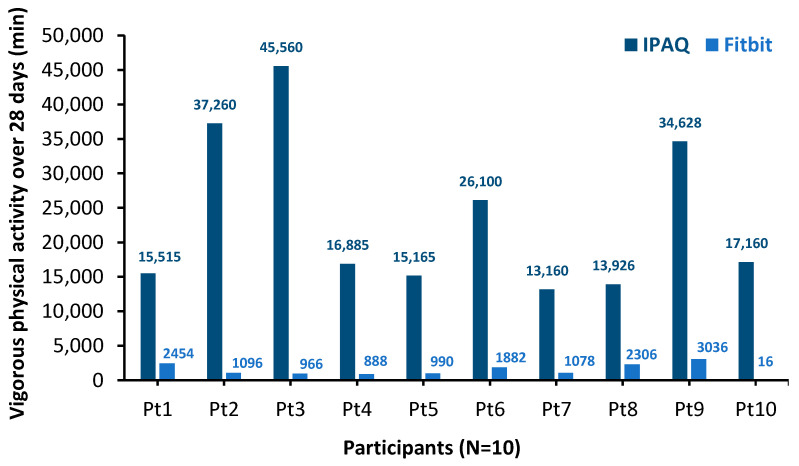
Minutes of vigorous physical activity accumulated over the 28-day study period by participant (Pt1–Pt10), measured with the International Physical Activity Questionnaire (IPAQ) and the Actigraph Fitbit Inspire 3. Values represent cumulative minutes over four consecutive weeks, with IPAQ self-reported and Fitbit device-recorded.

Participant vigorous physical activity values are shown in Figure 2. As commonly observed, the self-reported IPAQ overestimated vigorous activity compared with the device-based Fitbit. Although individual variability in physical activity between participants was observed (see Figure 2), there was no significant difference in mean physical activity for all participants based on IPAQ over the 28-day study period (mean score = 5883.9 ± 3224.8; 95% CI: 4852.4–6915.5; *p* = 0.75, 1-way ANOVA). Furthermore, no difference was observed using the Fitbit for moderate activity (mean score = 28.7 ± 14.8, 95% CI: 24.7–32.5; *p* = 0.48) or intense activity (median = 51.4 ± 28.6; 95% CI: 43.9–58.9; *p* = 0.26) over the 28-day period. There was also no significant difference in the level of physical activity as measured by IPAQ for participants in sequence A vs. sequence B (*p* = 0.65, ANOVA), in addition to activity measured using Fitbit (*p* = 0.44 for moderate activity and *p* = 0.13 for intense activity; ANOVA).

All participants attended the four scheduled visits and completed the GSRS, which assesses 15 gastrointestinal symptoms across seven graded response options ranging from “no discomfort at all” to “very severe discomfort.” GSRS evaluation confirmed an overall excellent tolerability to B-bNs supplementation. Only one participant reported mild loose stools during the supplementation week, a symptom not considered an adverse effect of B-bNs supplementation, as it had already been experienced by the same athlete in the past.

### 3.2. Effects of Product Intake on NO Pathway in Acute and Subacute Phases

**Figure 3:**
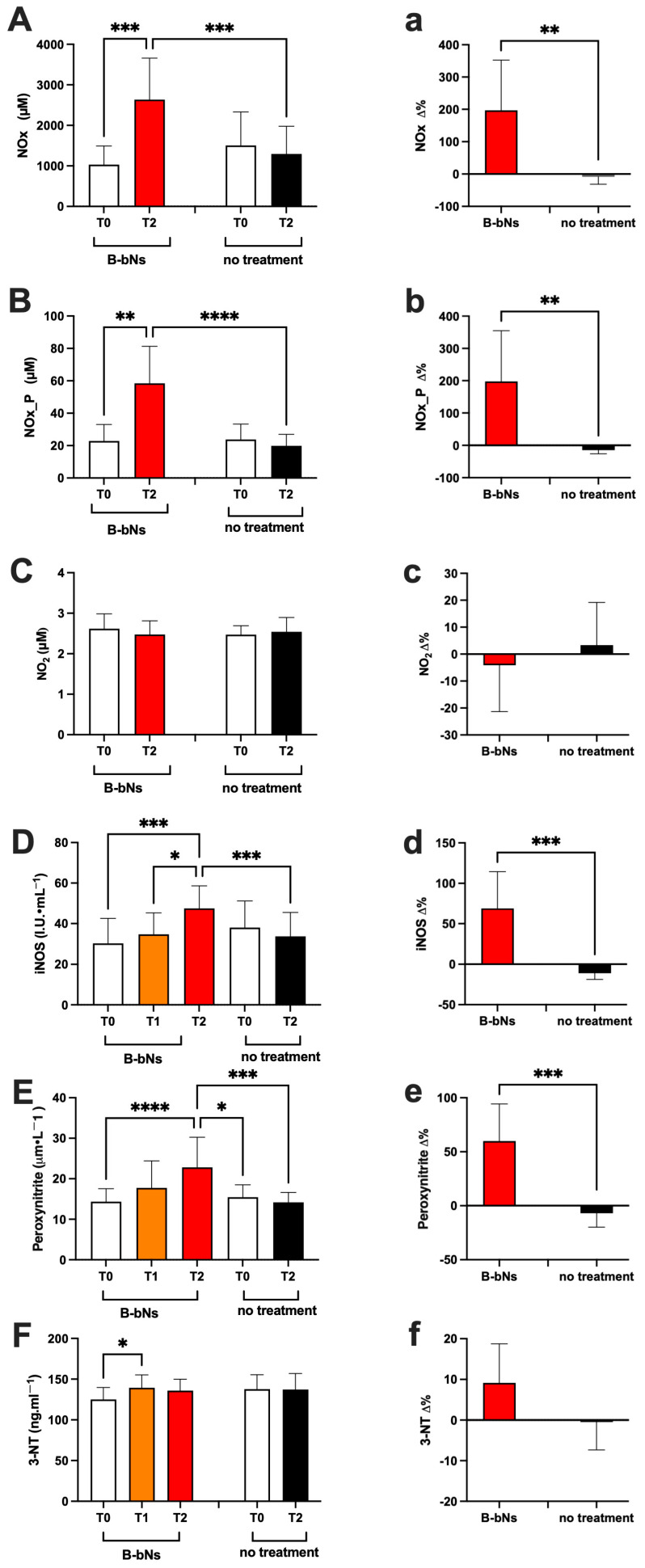
Absolute values (capital letter), and relative-variation (lowercase letter) at T0, T1 and T2 in beetroot-based nitrate supplementation (B-bNs), and T0 and T2 in no treatment groups: (**A**,**a**) NO metabolites in urine, (**B**,**b**) NO metabolites in plasma; (**C**,**c**) NO_2_ concentrations; (**D**,**d**) iNOS expression; (**E**,**e**) peroxynitrite; and (**F**,**f**) 3-NT concentrations. Statistically significant difference comparisons are displayed as: * *p* < 0.05; ** *p* < 0.01; *** *p* < 0.001, **** *p* < 0.0001.

Concerning the NO pathway, levels were assessed in plasma and/or urine. In detail, significant differences were calculated as follows:

**(i)** in treated groups (B-bNs) (A: Day 1–7 and B: 22–28):

NOx in urine (μM; Figure 3A,a) increased: T0 vs. T2: 1033 ± 457.3 vs. 2634 ± 1025; d-Cohen = 0.483; CI 95% 706.1–1360; 1901–3367; and similarly NOx in plasma increased (μM; Figure 3B,b): T0 vs. T2: 22.91 ± 10.15 vs. 58.49 ± 22.76; d-Cohen = 2.019; CI 95% 15.65–30.17; 42.20–74.77. Regarding NO_2_ concentrations in urine (μM; Figure 3C,c): no significant differences were found between T0 and T2 (T0: 2.61 ± 0.36; T2: 2.47 ± 0.33). iNOS expression in plasma increased significantly (IU·mL^−1^; Figure 3D,d): T0 vs. T1 and T2: 30.39 ± 12.18 vs. 34.69 ± 10.62 and vs. 47.50 ± 11.14; d-Cohen = 0.376, and 3.473; CI 95% 21.68–39.10; 27.09–42.28; 39.53–55.46. Peroxynitrite in plasma significantly increased (μm·L^−1^; Figure 3E,e): T0 vs. T2: 14.34 ± 3.21 vs. 22.82 ± 7.42; d-Cohen = 1.483; CI 95% 12.04–16.64; 17.51–28.13; similarly the 3-NT concentration in plasma increased (ng·mL^−1^; Figure 3F,f): T0 vs. T1: 125.1 ± 14.69 vs. 139.4 ± 15.88; d-Cohen = 0.934; CI 95% 114.6–135.6; 128.0–150.8.

**(ii)** in no treatment groups (A: Day 22–28 and B: 1–7):

NOx in urine and in plasma (μM; Figure 3A,a,B,b): no significant differences (NOx urine T0: 1504 ± 827.4; T2: 1296 ± 680.8; NOx plasma T0: 2.47 ± 0.21; T2: 2.54 ± 0.35) were found. Similar results with no significant differences between T0 and T2 were found for NO_2_ in urine (μM; Figure 3C,c T0: 2.47 ± 0.21; T2: 2.54 ± 0.35) and iNOS in plasma (IU·mL^−1^; Figure 3D,d; T0: 38.02 ± 13.14; T2: 33.73 ± 11.74). Plasmatic peroxynitrite (μm·L^−1^; Figure 3E,e; T0: 15.46 ± 3.06; T2: 14.19 ± 2.42) and 3-NT (ng·mL^−1^; Figure 3F,f T0: 137.8 ± 17.58; T2: 137.2 ± 19.77), showed no significant differences between T0 and T2.

**(iii)** inter-groups (B-bNs vs. no treatment):

NO metabolites were significantly higher at T2 in B-bNs groups, both in urine (μM; Figure 3A,a): 2634 ± 1504 vs. 1296 ± 680.8 (d-Cohen = 0.271; CI 95% 1901–3367; and 808.9–1783); and in plasma (μM; Figure 3B,b): 58.49 ± 22.76 vs. 19.85 ± 7.10 (d-Cohen = 2.399; CI 95% 42.20–74.77; and 14.76–42.13). The NO_2_ concentrations in urine (μM; Figure 3C,c) and 3-NT in plasma (ng·mL^−1^; Figure 3F,f) showed no significant inter-group differences. Significantly lower plasmatic values at T2 in no treated vs. B-bNs groups both iNOS (IU·mL^−1^; Figure 3D,d): 33.73 ± 11.74 vs. 47.50 ± 11.14 (d-Cohen = 1.203; CI 95% 25.34–24.93 and 39.53–55.46) and peroxynitrite (μm·L^−1^; Figure 3E,e): 22.82 ± 7.42 vs. 15.46 ± 3.06 and vs. 14.19 ± 2.42; (d-Cohen = 1.296, and 1.563; CI 95% 17.51–28.13; vs. 13.27–17.65 and 12.46–15.93) were found.

### 3.3. Effects of Product Intake on Oxy-Inflammation in Acute and Subacute Phases

**Figure 4:**
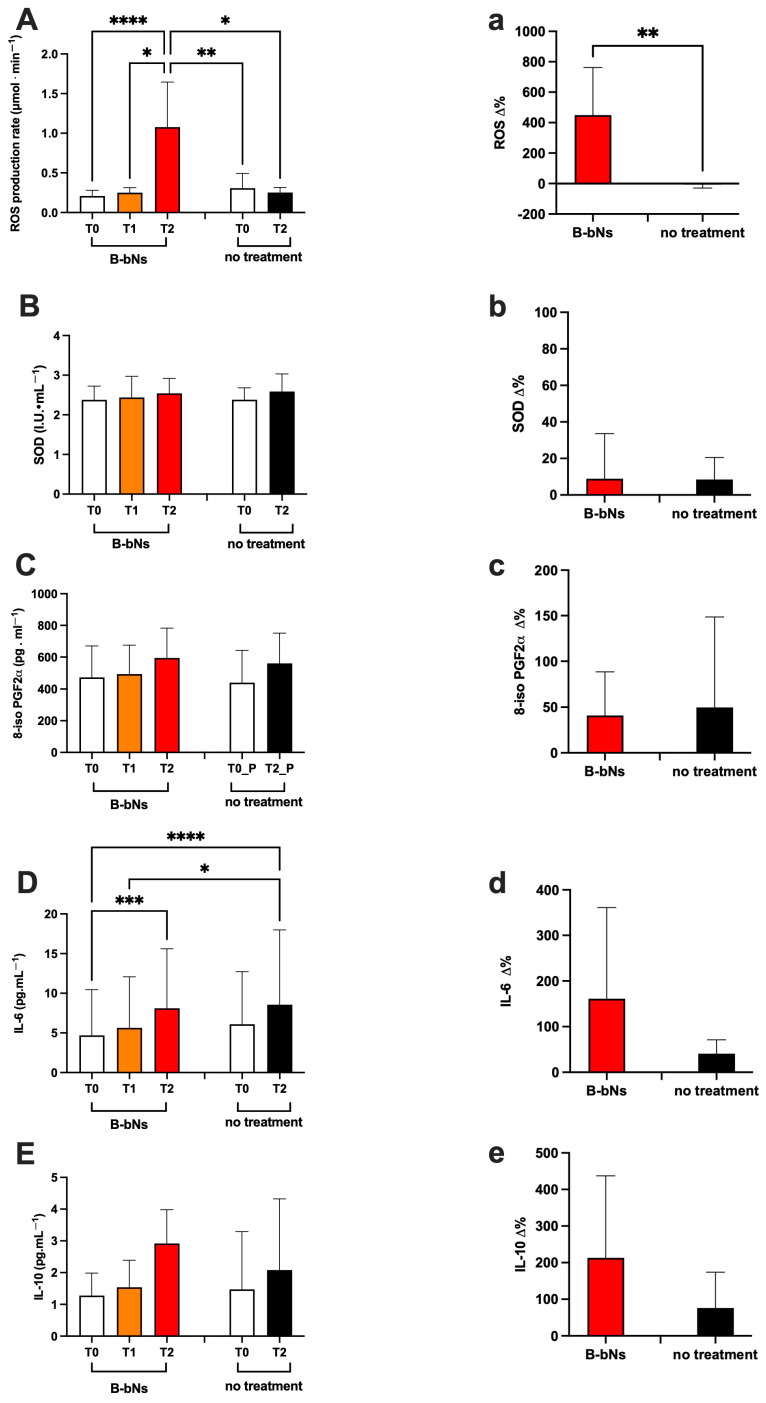
Absolute values (capital letter), and relative-variation (lowercase letter) at T0, T1 and T2 in beetroot-based nitrate supplementation (B.bNs), and T0 and T2 in no treatment groups: (**A**,**a**) ROS production rate, (**B**,**b**) SOD activity; (**C**,**c**) 8-iso-PGF2α concentration; (**D**,**d**) IL-6 levels; (**E**,**e**) IL-10 levels. Statistically significant difference comparisons are displayed as: * *p* < 0.05; ** *p* < 0.01; *** *p* < 0.001; **** *p* < 0.0001.

The ROS production rate, the inflammatory status (levels of IL-6 and IL-10), and SOD activity in plasma and biomarker concentration of lipid peroxidation (8-iso-PGF2α) in urine, assessed in subjects pre and after treatment or no-treatment periods, are displayed in Figure 4.

The statistically significant differences, intra and inter-group, calculated from the data collected between different groups, are herein reported. In detail, significant differences were calculated as follows:

**(i)** in treated groups (B-bNs) (A: Day 1–7 and B: 22–28):

ROS production (μmol·min^−1^; Figure 4A,a) significantly increased: T0 vs. T2 0.209 ± 0.07 vs. 1.07 ± 0.56; d-Cohen = 0.613; CI 95% 0.15–0.26; 0.20–0.29; and T1 vs. T2 0.250 ± 0.063 vs. 1.07 ± 0.56; d-Cohen = 2.079; CI 95% 0.20–0.29; 0.67–1.48. SOD activity (U·mL^−1^; Figure 4B,b): no significant differences (T0: 2.380 ± 0.345; T1: 2.437 ± 0.533; T2: 2.541 ± 0.378); 8-iso-PGF2α (pg·mL^−1^; Figure 4C,c): no significant differences (T0: 472.2 ± 198.3; T1: 493.1 ± 182.6; T2: 594.8 ± 188.3) were shown. IL-6 (pg·mL^−1^; Figure 4D,d) showed a significant increase: T0 vs. T2: 4.69 ± 5.76 vs. 8.11 ± 7.50; d-Cohen = 4.105, and 2.163; CI 95% 0.57–8.82; 2.74–13.48; IL-10 (pg·mL^−1^; Figure 4E,e): no significant differences (T0 vs. T1 and vs. T2: 1.27 ± 0.71 vs. 1.54 ± 0.84 and vs. 2.91 ± 1.06) were shown.

**(ii)** in no-treatment groups (A: Day 22–28 and B: 1–7):

ROS (μmol·min^−1^; Figure 4A,a): no significant differences (T0: 0.308 ± 0.184; T2: 0.252 ± 0.063); SOD (U·mL^−1^; Figure 4B,b): no significant differences (T0: 2.382 ± 0.298; T2: 2.587 ± 0.443); 8-iso-PGF2α (pg·mL^−1^; Figure 4C,c): no significant differences (T0: 438.8 ± 203.5; T2: 560.4 ± 190.9); IL-6 (pg·mL^−1^; Figure 4D,d): no significant differences (T0: 6.09 ± 6.63; T2: 8.56 ± 9.43); IL-10 (pg·mL^−1^; Figure 4E,e): no significant differences (T0: 1.47 ± 1.82; T2: 2.08 ± 2.24) were shown.

**(iii)** inter-groups (B-bNs vs. no treatment): ROS (μmol·min^−1^; Figure 4A,a) significantly different: B-bNs T2 vs. no treatment T0 and T2: 1.078 ± 0.568 vs. 0.308 ± 0.184 and 0.252 ± 0.063; d-Cohen = 1.832, and 2.044; CI 95% 0.67–1.48; 0.17–0.43; 0.20–0.29 IL-6 (pg·mL^−1^; Figure 4E,e) significantly different: B-bNs T0 vs. no treatment T2. 4.69 ± 5.76 vs. 8.56 ± 9.43; d-Cohen = 0.495; CI 95% 0.57–8.82; 1.81–15.31; and B-bNs T1 vs. no treatment T2: 5.64 ± 6.43 vs. 8.56 ± 9.43; d-Cohen = 0.361; CI 95% 1.04–10.24; 1.81–15.31; SOD, 8-iso-PGF2α and IL-10 no significant differences were shown.

## 4. Discussion

In this randomized crossover study, our findings suggest that a B-bNs supplementation could effectively modulate key biochemical pathways linked to NO metabolism, redox balance, and inflammation in trained elite triathletes. The observed pattern indicates evidence of enhanced NO bioavailability and nitric-peroxide redox activation, occurring without overt evidence of oxidative damage to lipids. These preliminary findings support the effects on the NO-related pathway and safety of dietary nitrate supplementation in endurance-trained individuals. Although the short- and long-term (chronic) effects of nitrate intake have raised concerns regarding the potential formation of *N-*nitrosate compounds, the associated risk from plant-derived sources is believed to be lower than that from industrial food nitrates [57].

Regarding the safety of beet root consumption, a pilot study investigated the effects of two-week consumption on gut microbiota modulation. In that study, the red beetroot intake can influence gut microbial composition and associated catabolites, suggesting potential implications for intestinal and metabolic health [34]. The detection of beetroot-derived betalains and their metabolites in stool supported the involvement of these compounds in microbe–host interactions. As per available literature, no specific adverse health effects have been associated with BJ supplementation, except for episodes of gastrointestinal illness linked to raw, unprocessed grated beetroot, likely due to microbial contamination and unrelated to processed juice products [35].

The present group of endurance-trained elite male triathletes, with normal BMI and low adiposity, provides an appropriate model for testing nitrate-rich beetroot formulations. Notably, B-bNs were well tolerated, with no gastrointestinal or systemic adverse events reported throughout the intervention, consistent with evidence that beetroot-derived nitrate is generally safe in humans, including athletes, when used within physiological dosing ranges [58,59,60,61].

Consistent with appropriate randomization, baseline biomarker distributions were largely comparable between the B-bNs and the no-treatment conditions. Only NOx and 3-NT were observed to be modestly higher in the no-treatment data at baseline. These small differences are unlikely to be attributable to the pooling of data from the two groups. Indeed, because the follow-up period in group A was not sufficiently long, the data recorded at visit 3 in this group were higher than those recorded in group B prior to the no-treatment period. Nevertheless, we are confident that the changes observed after supplementation reflect genuine treatment-related effects.

Following acute ingestion, plasma iNOS and peroxynitrite concentrations increased significantly, indicating rapid activation of the NO pathway and downstream nitrative activity. Peroxynitrite, a reactive nitrogen species formed by the reaction of NO with superoxide, serves as both an effector and a marker of NO bioactivity, reflecting the balance between NO signaling and redox flux [62]. The formation of peroxynitrite is faster than the scavenging of superoxide by SOD [63], so even if SOD activity was unchanged, it cannot compete for the superoxide produced that reacts with NO generated by supplementation. Therefore, peroxynitrite concentration quickly increased. The associated rise in 3-NT provides biochemical evidence of protein nitration, a stable footprint of peroxynitrite interaction with tyrosine residues and is thus indicative of enhanced NO-derived signaling rather than pathological nitrative stress at the levels observed [64,65]. After seven days, elevated NOx and iNOS persisted, suggesting sustained NO generation under continued supplementation. This pattern is consistent with reports that nitrate ingestion leads to maintained elevations in circulating NO metabolites and improved NO-dependent physiological responses [66,67,68].

Importantly, although these increases in NOx, iNOS, peroxynitrite, ROS, IL-6, and 3-NT may suggest activation of inflammatory and nitro-oxidative pathways, this profile is consistent with the well-described acute response to enhanced NO metabolism following nitrate supplementation [68,69,70,71]. In this regard, these mediators are increasingly recognized as components of transient redox and signaling activation rather than indicators of pathological stress. Reactive oxygen and nitrogen species, together with cytokines such as IL-6, play regulatory roles in vascular, metabolic, and immune signaling, particularly in trained individuals, where controlled elevations contribute to adaptive processes [72,73].

Moreover, increased NO bioavailability is associated with improved endothelial function, vasodilation, and blood pressure regulation [74,75,76,77]. Therefore, the observed pattern likely reflects a short-term physiological response rather than a deleterious or pro-inflammatory condition, particularly in the absence of increased lipid peroxidation markers. Taken together, these results suggest that B-bNs activates both the canonical NO synthesis (via iNOS) and the nitrate–nitrite–NO reduction pathway, accompanied by mild, physiologically relevant nitrative activity as part of normal downstream signaling.

**Figure 5:**
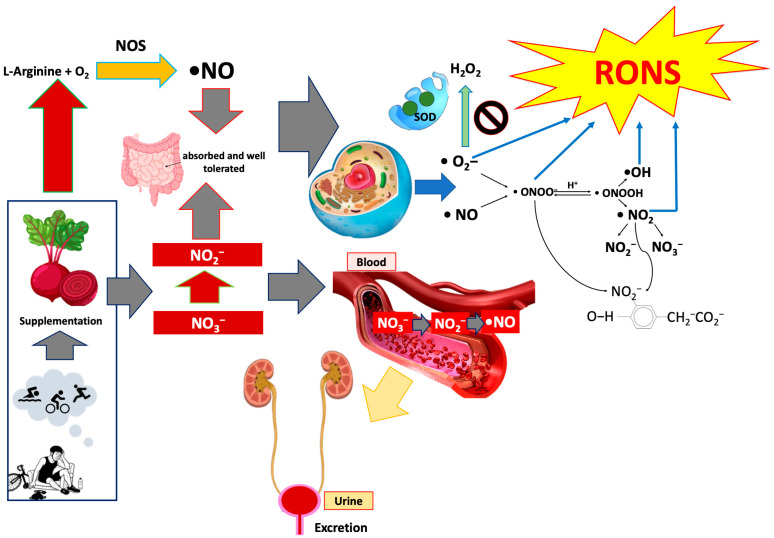
Schematic diagram of the mechanisms underlying beetroot-based nitrate supplementation (B-bNs) and its effects on nitric oxide metabolism, redox balance, and inflammation in endurance athletes. Dietary nitrate (NO_3_^−^) is absorbed and reduced to nitrite (NO_2_^−^) and nitric oxide (NO), increasing systemic NO bioavailability in blood and urine. Enhanced NO signaling is associated with increased nitric oxide synthase (NOS) activity and downstream formation of reactive nitrogen species, including peroxynitrite and 3-nitrotyrosine. Although reactive oxygen species production rises, antioxidant defenses (e.g., SOD activity) and lipid peroxidation markers remain unchanged, indicating preserved redox balance. Mild increases in IL-6 reflect physiological adaptation rather than pathological inflammation, supporting the safety and efficacy of B-bNs in modulating NO-related pathways without inducing oxidative damage. •NO, nitric oxide; H_2_O_2_, hydrogen peroxide; NOS, nitric oxide synthase; NO_2_^−^, nitrite; NO_3_^−^, nitrate; NO_2_, nitrogen dioxide; NO_2_^−^/NO_3_^−^, nitrite/nitrate pool; ONOO^−^, peroxynitrite; ONOOH, peroxynitrous acid; O_2_^−^, superoxide anion; •OH, hydroxyl radical; RONS, reactive oxygen and nitrogen species; SOD, superoxide dismutase.

Alongside nitrative activation, acute B-bNs intake induced a moderate rise in 8-iso-PGF_2_α. ROS generation is an expected consequence of enhanced NO flux (Figure 5) that generates its free radicals. The formation of 8-iso-PGF_2_α, a non-enzymatic lipid peroxidation product, reflects transient membrane oxidative turnover and is often used as a sensitive indicator of oxidative–inflammatory cross-talk [14]. In some studies, an increase in lipid peroxidation with beetroot supplementation in trained athletes was observed [7]. The modest, short-lived increase in 8-iso-PGF_2_α observed here suggests an adaptive redox activation rather than cellular damage. After seven days of supplementation, ROS remained elevated, while 8-iso-PGF_2_α was stabilized, indicating that the redox system had reached a new steady state characterized by higher signaling activity but controlled oxidative output. This dissociation suggests that the initial, damaging ROS bursts from a stimulus (like exercise or nitrate supplementation) triggered enhanced antioxidant capacity and mitochondrial efficiency, leading to a controlled redox state that uses ROS for signaling rather than causing widespread damage. These findings mirror previous ones in nitrate supplementation and exercise studies, where transient ROS bursts enhance antioxidant capacity and mitochondrial efficiency [78,79,80,81]. Collectively, these observations support the interpretation that B-bNs induce a controlled redox response, stimulating redox-sensitive signaling cascades without progressing into oxidative damage.

The concurrent increases in IL-6 and IL-10 observed after both acute and subacute supplementation indicate an immune response linked to NO and redox activation. IL-6, widely recognized as a myokine, is released during metabolic and vascular stimulation to promote substrate mobilization and angiogenesis [82], while IL-10 serves as an anti-inflammatory regulator that limits cytokine overactivation [15]. The concurrent upregulation of these cytokines thus reflects an integrated redox-immune adaptation rather than inflammation per se. Notably, ROS and 8-iso-PGF_2_α are known to act as redox messengers in the activation of NF-κB and other transcription factors regulating IL-6 production [83], providing a mechanistic link between oxidative signaling and cytokine modulation. After seven days, both IL-6 and IL-10 remained modestly elevated but did not diverge between conditions, suggesting that immune activation was transient and self-limiting. Similar redox-linked cytokine profiles have been reported after nitrate or beetroot supplementation and endurance exercise [84,85].

The increases in NOx, iNOS, and peroxynitrite observed in this study may reflect controlled redox priming that enhances signaling without shifting the redox system toward damage of lipids. Such activation may be physiologically beneficial, as mild ROS generation can trigger adaptive mitochondrial and vascular responses that improve oxygen delivery and muscle contractility [66,86]. Despite elevated NO-related and redox-active species, B-bNs was not observed to affect biomarkers of lipid peroxidation, consistent with evidence that beetroot-derived nitrate supplementation enhances NO signaling while preserving redox homeostasis. In clinical studies, beetroot intake reduced IL-6 and TNF-α without affecting MDA or hs-CRP in type 2 diabetes [87], improved redox balance in hypertension [31], and enhanced muscle performance in older adults without increasing oxidative damage markers [88]. Similar findings have been reported in athletes, with improved recovery and reduced DOMS without changes in muscle damage or inflammatory markers [89], and a systematic review in elite soccer players showing performance benefits without pro-oxidative effects [26].

## 5. Study Limitations

This study has some methodological limitations that should be considered when interpreting the findings. The sample size was limited, as the investigation was conducted in a small cohort of well-trained athletes. Due to the small sample size, our study was not sufficiently powered to evaluate causality nor adjust for potential confounding variables through covariate-adjusted models (e.g., ANCOVA or multivariate regression). We used a 15-day washout period between interventions. However, given the exploratory nature and limited sample size of this pilot study, it was not specifically powered to formally detect carryover effects.

Furthermore, the inclusion of only male participants limits the generalizability of our findings. In addition, the mean age of the cohort (~48 years) may influence NO bioavailability and vascular responses, and therefore the results may not be directly applicable to younger athletic populations. Furthermore, the open-label design may have introduced potential bias; however, the primary outcomes were based on objective biochemical measurements, which are less susceptible to expectancy effects.

Physical activity and training load were monitored using wearable devices and self-reported questionnaires; however, these methods may not have fully captured exercise intensity and physiological stress, which are particularly relevant in endurance-trained triathletes, as training load strongly influences NO-, redox-, and cytokine-related biomarkers. Dietary intake during the intervention was not standardized or recorded beyond a baseline food frequency questionnaire, limiting the ability to account for variability in dietary nitrate and antioxidant intake.

An additional limitation relates to the body weight-based dosing strategy, which used broad categories and may have introduced variability between participants with similar body weights. Although this effect was likely limited due to the relatively homogeneous cohort, this stepwise approach may have influenced inter-individual responses to nitrate exposure.

It should also be acknowledged that in addition to nitrate derived from beetroot extract, the supplement also contained other bioactive compounds (e.g., L-citrulline, L-arginine, and *N-*acetylcysteine) that may contribute to nitric oxide metabolism and redox regulation through complementary pathways [17,68,90,91]. Therefore, the observed effects may also reflect the combined action of these ingredients rather than nitrate alone.

In addition, baseline differences between sequences, the timing of the crossover phases, and circadian variability may have contributed to biomarker fluctuations. Finally, the absence of additional antioxidant markers and the lack of physiological or performance outcomes restrict the interpretation of the presented biochemical findings.

Regardless, the present investigation was designed as a pilot exploratory study, primarily aimed at assessing feasibility and generating preliminary data to guide larger controlled trials. In this regard, sample sizes comparable to or even smaller than ours are commonly reported in the literature investigating the physiological effects of dietary nitrate or beetroot supplementation [92,93]. While this design reduces inter-individual variability and allows for precise biochemical monitoring, larger studies are warranted to confirm these results across broader athletic populations. The intervention period was relatively short (7 days), focusing on acute redox and NO responses rather than long-term adaptations. In addition, the follow-up duration was insufficient to determine whether the observed biochemical changes persist or adapt after discontinuation of supplementation.

Nonetheless, the observed pattern, selective NO-redox activation without oxidative damage to lipids, provides valuable mechanistic insight into the early biochemical effects of nitrate supplementation. Although the present analysis evaluated molecular and redox biomarkers, functional or performance-related measures were not the primary endpoints of this study, and future studies will be essential to better translate these molecular findings into practical implications for endurance performance and recovery.

Future studies with longer follow-up periods, using precise weight-adjusted dosing and integrating physiological and performance outcomes, would help to clarify the practical implications of these biochemical changes. Dietary nitrate intake and oral microbiota composition, both known modulators of nitrate metabolism, were not directly quantified, but all participants followed standardized dietary guidelines and maintained stable training routines throughout the study to minimize variability.

## 6. Study Strengths

This study presents several strengths that should be considered alongside its limitations. First, this pilot investigation was undertaken in a relatively homogeneous cohort of well-trained amateur triathletes, with similar age, sex, training background, and body composition. This reduces inter-individual variability and internal consistency of the observed biochemical responses.

Second, the randomized 2 × 2 cross-over design represents an important methodological strength, as each participant served as his own control. This approach improves the ability to detect intervention-related effects despite the limited sample size and is particularly suitable for exploratory physiological studies.

A key strength was the comprehensive assessment of biomarkers spanning nitric oxide metabolism, oxidative stress, and inflammatory pathways. By simultaneously evaluating NOx, iNOS, peroxynitrite, 3-nitrotyrosine, ROS production, SOD activity, 8-isoprostane, and cytokines (IL-6 and IL-10), the study provides an integrated view of NO-redox-immune interactions, which is rarely captured within a single design.

In addition, the inclusion of both acute (2 h) and subacute (7-day) time points provides information on the temporal change in nitrate-induced responses.

Finally, the use of objective biochemical endpoints, including advanced techniques such as EPR for ROS measurement, strengthens the robustness of our findings and limits the influence of subjective bias.

## 7. Conclusions

Taken together, our findings suggest that B-bNs may produce a dual effect: enhancement of NO-mediated vasodilatory and metabolic signaling, and preservation of oxidative homeostasis. The simultaneous increases in iNOS, ROS, and peroxynitrite may likely reflect adaptive rather than deleterious oxidative signaling. Such controlled activation may optimize muscle perfusion, oxygen utilization, and mitochondrial efficiency, ultimately contributing to improved performance and recovery potential.

From a physiological perspective, the observed increase in NO-related metabolites may have relevant implications for endurance exercise. Enhanced NO bioavailability is known to promote vasodilation, thereby improving muscle perfusion and oxygen delivery during exercise, as well as reducing the oxygen cost of submaximal work.

In addition, NO has been shown to influence mitochondrial efficiency and contractile function, potentially contributing to improved exercise tolerance and delayed fatigue [94,95,96]. From a practical perspective, these findings suggest that nitrate supplementation may support redox balance and NO-related signaling in endurance-trained individuals, potentially contributing to more efficient physiological responses to training. Although performance outcomes were not directly assessed, the observed biochemical adaptations may have relevance for exercise tolerance and recovery. However, these implications should be interpreted with caution and confirmed in future studies integrating both molecular and performance-based outcomes.

The absence of elevated lipid peroxidation may underline the safety and physiological compatibility of this intervention. Importantly, beetroot-derived antioxidants (betalains, polyphenols, ascorbate) likely buffer potential ROS excess, maintaining the redox balance within a safe range. This endogenous buffering may explain why in B-bNs, enhanced NO/redox activity occurs without provoking oxidative damage, consistent with previous reports that beetroot supplementation strengthens the antioxidant defense system [80].

## Figures and Tables

**Table 1:** Dose of Nobeet^®^ administered to each participant according to body weight.

Participant ID	Dose (g)	Body Weight (kg)
1	20	81.3
2	10	70.3
4	10	79.8
5	10	64.4
6	10	61.8
7	10	79.9
8	10	65.1
9	10	75.3
10	10	70.8
11	30	94.3

**Table 2:** Baseline characteristics of participants.

Characteristic	*N* = 10
Age (years)	48.1 ± 9.8
Weight (kg)	74.4 ± 9.5
Height (cm)	175.3 ± 9.1
BMI (kg/m^2^)	23.9 ± 2.2
Body fat (%)	10.1 ± 4.9
Fat mass (kg)	7.8 ± 5.1
Fat-free mass (%)	66.6 ± 6.7
Total body water (kg)	48.8 ± 5.0

Data are presented as mean and standard deviation. BMI = body mass index.

## Data Availability

The original contributions presented in this study are included in the article. Further inquiries can be directed to the corresponding author.

## Abbreviations

B-bNs	Beetroot-based nitrate supplement
CMH	1-hydroxy-3-methoxy-carbonyl-2,2,5,5-tetramethylpyrrolidine
CP	3-carboxy2,2,5,5- tetramethyl1-1-pyrrolidi-nyloxy
EPR	Electron paramagnetic resonance spectroscopy
IL-6	Interleukin-6
IL-10	Interleukin-10
iNOS	Inducible nitric oxide synthase
NOx	Nitric oxide metabolites
RON	Reactive oxygen and nitrogen species
ROS	Reactive oxygen species
SOD	Superoxide dismutase
3-NT	Nitrotyrosine
8-iso-PGF2α	8-iso-prostaglandin F2α
